# Is the Antimicrobial Activity of Hydrolates Lower than that of Essential Oils?

**DOI:** 10.3390/antibiotics10010088

**Published:** 2021-01-18

**Authors:** Maura Di Vito, Antonina Smolka, Maria Rita Proto, Lorenzo Barbanti, Fabrizio Gelmini, Edoardo Napoli, Maria Grazia Bellardi, Paola Mattarelli, Giangiacomo Beretta, Maurizio Sanguinetti, Francesca Bugli

**Affiliations:** 1 Dipartimento di Scienze e Tecnologie Agro-Alimentari, Università of Bologna, Viale G. Fanin 42, 40127 Bologna, Italy; mariarita.proto2@unibo.it (M.R.P.); lorenzo.barbanti@unibo.it (L.B.); mariagrazia.bellardi@unibo.it (M.G.B.); paola.mattarelli@unibo.it (P.M.); 2 Dipartimento di Scienze Biotecnologiche di Base, Cliniche Intensivologiche e Perioperatorie, Università Cattolica del Sacro Cuore, Largo A. Gemelli 8, 00168 Rome, Italy; smolka2015@gmail.com (A.S.); maurizio.sanguinetti@unicatt.it (M.S.); francesca.bugli@unicatt.it (F.B.); 3 Dipartimento di Scienze e Politiche Ambientali, Università degli Studi di Milano, via Celoria, 2, 20133 Milano, Italy; fabrizio.gelmini@unimi.it (F.G.); giangiacomo.beretta@unimi.it (G.B.); 4 Istituto di Chimica Biomolecolare, Consiglio Nazionale delle Ricerche, via P. Gaifami 18, 95126 Catania, Italy; edoardo.napoli@icb.cnr.it; 5 Dipartimento di Scienze di Laboratorio e Infettivologiche, Fondazione Policlinico Universitario “A. Gemelli” IRCCS, 00168 Rome, Italy

**Keywords:** *Satureja montana*, *Lavandula angustifolia*, *Lavandula intermedia*, *Origanum hirtum*, *Monarda didyma*, *Monarda fistulosa*

## Abstract

Among the top five human infections requiring medical treatment is dermatitis. Treatment of bacterial and fungal skin infections is usually based on antibiotic therapy, which is often ineffective due to the involvement of antibiotic-resistant microbial strains. The aim of this study was to compare the antimicrobial activity of essential oils (EOs) and hydrolates (Hys) extracted from six aromatic plants grown in Italy (*Lavandula angustifolia, Lavandula intermedia, Origanum hirtum, Satureja montana, Monarda didyma,* and *Monarda fistulosa*) towards fungal (*Candida albicans*, *Candida parapsilosis*, *Candida glabrata* and *Candida tropicalis*; *Trichophyton soudanense*, *Trichophyton tonsurans*, *Trichophyton rubrum*, *Trichophyton violaceum* and *Microsporum canis*) and bacterial strains (*Staphylococcus aureus* MRSA, *Staphylococcus aureus* MSSA, *Streptococcus pyogenes*, *E. faecalis, Enterococcus faecalis* VRE, and *Enterococcus faecium*) potentially pathogenic for human skin. The composition and antimicrobial activity of EOs and Hys were evaluated using the Gas-chromatography mass spectrometry and micro dilution-broth test, respectively. The volatiles’ conversion factors (CFs) were calculated to compare the activity of Hys with that of the corresponding EOs. Data show that, although the minimum inhibitory concentration values of EOs are lower than the corresponding Hys, the volatiles contained in Hys are more effective at inhibiting microbial growth because they are active at lower concentrations.

## 1. Introduction

Among the top five human infections requiring medical treatment is dermatitis [1]. Treatment of bacterial and fungal skin infections is usually based on antibiotic therapy, which is often ineffective due to the involvement of antibiotic-resistant microbial strains such as methicillin-resistant *Staphylococcus aureus* (MRSA) [2] and *Candida* sp. [3]. In recent decades, given the poor innovation in the discovery of new antimicrobials and the frequency of recalcitrant skin infections, the need for innovative anti-infective therapeutics is becoming more and more urgent. In this field, great interest in the last 20 years has been focused on the potential of natural products.

In recent years, there has been growing interest in natural products obtained from aromatic plant distillation: essential oils (EOs) and hydrolates (Hys). As such, there are many scientific articles about the effectiveness of EOs in various contexts: antimicrobials, immunomodulatory, antioxidants, anti-inflammatory, pain-relievers, etc., but there is little evidence on the activities of Hys.

Official Pharmacopoeias well define the two natural products. The EO is considered to be a complex odorous product obtained by steam distillation, hydro-distillation, or by the dry distillation of a plant, some of its parts, or, in the case of OEs obtained from *Citrus* spp., through appropriate mechanical cold processes [4]. Similarly, starting from 2012, the French Pharmacopoeia defines the Hy as a product obtained through the distillation of different parts of aromatic plants, which separates from the essential oil at the end of the distillation [5].

While they originate from the same process, the two distillation products are quite different in terms of chemical composition and effectiveness.

EOs are hydrophobic mixtures mainly characterized by terpene molecules that, on the contrary, are extremely diluted in Hys. In fact, the Hys are hydrophilic solutions characterized, up to a maximum of 1 g/L, by the terpene components present in the corresponding EO [6]. Furthermore, in the Hy, the relative ratio of each terpenic molecule will be conditioned by its hydrophilic characteristics. Owing to this, the major components of an EO may not be the same that is present in the corresponding Hy.

Due to the high oxicity of many terpene compounds [7], essential oils require special warnings when used *per os* or in topical applications [8]. On the contrary, Hys resulting from dilution of terpenic solutions are less toxic and can be used more easily for the same applications.

However, only few studies have been carried out on EOs and Hys obtained from the same distillation process in order to compare their chemical composition [9,10,11], or study some of their activities such as psychopharmacological and anti-cancer activities [12,13], or larvicidal and nematodicidal ones [14,15]. Our group participated in these early investigations, assessing the chemical composition and the antimicrobial activity of the EO and Hy obtained from *Monarda citriodora* in a recent research. The study showed that, to achieve the same inhibitory effect of EO, a higher volume of Hy was necessary; however, in this volume, the concentration of active components was lower than that present in the corresponding EO, i.e., the EO from the same plant source [16]. Therefore, data indicate a higher likelihood for the active compounds isolated from *M. citriodora* Hy to be more active in the aqueous phase, because they can more easily reach their target, or because they are not contrasted with antagonistic compounds present only in the OE.

Given this background and in view of improving the knowledge on Hy potential uses, the first aim of this study was to evaluate the antimicrobial activity of six EOs and the companion Hys isolated from the same aromatic plant cultivated in Italy, towards fungal and bacterial strains potentially pathogenic for human skin. The following microorganisms isolated from patients with skin infections included the following. Six bacteria: methicillin-resistant *Staphylococcus aureus* (MRSA), methicillin- susceptible *Staphylococcus aureus* (MSSA), *Streptococcus pyogenes*, vancomycin-resistant enterococci (VRE) *Enterococcus faecalis* and *Enterococcus faecium*. Four drug-resistant yeasts: *Candida albicans*, *Candida parapsilosis*, *Candida glabrata* and *Candida tropicalis*. Five dermatophytes: *Trichophyton soudanense*, *Trichophyton tonsurans*, *Trichophyton rubrum*, *Trichophyton violaceum* and *Microsporum canis*. The second aim was to compare the relative concentration of active volatiles present in EOs and Hys obtained from the same plant by using the volatiles’ conversion factor (CF).

## 2. Results

### 2.1. GC-MS and Gravimetric Analyses

The chromatographic analysis of EOs shows phytocomplexes that are quite different (Table 1). *Lavandula angustifolia* has linalyl acetate and β-linalool at respective concentrations of 33.35% and 28.36%, while *L. intermedia* EO has the same components at concentrations of 36.47% and 27.99%, respectively. The EO of *Origanum hirtum* is mainly characterized by thymol, γ-terpinene and p-cymene at 36.3%, 23.81% and 18.83%, respectively, while the EO of *Satureja montana* has carvacrol as a major compound (concentration of 63.1%), followed by γ-terpinene (concentration of 13.44%). Both *Monarda didyma* and *M. fistulosa* EOs show carvacrol (20.59% and 35.18%, respectively) and γ-terpinene (13.07% and 16.85%, respectively) as major compounds, while thymol and *p*-cymene are the third most concentrated components in the respective *M. didyma* and *M. fistulosa*. The rest of the components present in EOs show concentrations lower than 10%.

The analysis of Hy (Table 2) shows β-linalool, α-terpinen-4-ol and α-terpineol (42.5%, 20.33 and 19.1%, respectively) as major chemical compounds of *L. angustifolia* Hy. *L. intermedia* Hy is characterized by β-linalool, camphor and 1,8-cineol (34.17%, 22.12% and 19.08%, respectively) as major compounds, while *S. montana* has carvacrol and thymol as the major compounds (85.79% and 13.88%, respectively). *O. hirtum* Hy has only one component, thymol (100% concentration).

*M. didyma* has carvacrol and thymol (48.44% and 34.03%, respectively) as major compounds, while *M. fistulosa* has only carvacrol (84.68%) at a concentration above 10%. All the other components show a concentration lower than 10%. It is important to remember that the concentrations of chemicals identified in the Hys are referred at most to 1 g/L, which is the maximum terpenes concentration present in Hy. Results of the gravimetric analyses are shown in Table 2. The qualitative and quantitative analyses of the extract obtained for the gravimetric analysis are not shown because they are redundant and perfectly superimposable to those obtained from the gas-chromatographic analysis.

### 2.2. Broth Microdilution Susceptibility Test

Table 3 shows the Minimum Inhibitory Concentration (MIC) and Minimum Lethal Concentration (MLC) of the tested EOs. The table also displays the values of Inhibition Rate or Lethal Rate of 90% (IR90 and LR90, respectively) of strains. The EOs of *S. montana* and *O. hirtum* are the most active, showing IR90 values of 0.25% and 1 % *v*/*v*, respectively, and LR90 values of 0.25% *v*/*v* and 1% *v*/*v*, respectively. All the other EOs have IR90 and LR90 values greater than or equal to 2% *v*/*v*, except *M. didyma* EO showing IR90 and LR90 values equal to 1% *v*/*v* and > 2% *v*/*v*, respectively. Specifically, while the EO of *S. montana* acts in equal measure on all three microbial types (bacteria, yeasts, and dermatophytes), the EO of *O. hirtum* acts primarily on bacteria and yeasts, while that of *M. fistulosa* on dermatophytes.

As shown in Table 4, values obtained from the analysis of the antimicrobial effectiveness of the Hys indicate the Hys of *O. hirtum* and *M. didyma* (IR90 value 50% *v*/*v*) as more active than the others against bacteria, yeast and dermatophytes. However, it was not possible to study Hys concentrations greater than 50% *v*/*v*, as this would have introduced a significant methodological bias by reducing the amount of nutrient broth necessary for microbial growth.

In particular, the *O. hirtum* Hy at a concentration of 50% *v*/*v* is the only one that can inhibit all bacteria growth but is unable to exert cytocidal effect at the same concentration, while fungi (yeast and dermatophytes) show greater sensitivity to Hys (Table 3). Specifically, the Hys of *S. montana*, *O. hirtum* and *M. didyma* have inhibitory and cytocidal effect against most dermatophytes at a concentration equal to 50% *v*/*v*, and only *M. fistulosa* is able to inhibit all strains at a concentration of 25% *v*/*v,* but it is not capable of having cytocidal effects for values <50% *v*/*v*.

### 2.3. Comparison Between EOs and Hys

Table 5 shows the values of the peaks’ total areas of the chemicals of both EOs (EOTA) and Hys (HYTA), the volatiles’ Conversion Factor (CF) obtained as EOTA/ HYTA, and the value of the IR50_Hy_/CF ratio. This last parameter indicates the value that the IR50_Hy_ would have if the Hy were concentrated as the EO. As shown in Table 5, the value of the IR50_Hy_/CF ratio is lower than that of IR50_E_o for all the EOs.

This means that, to have the same antimicrobial activity in the EO, a relative concentration of volatiles between 1.11 (*S. montana*) and 71.43 (*M. fistulosa*) times as high as that contained in the Hy is required.

The same difference is evidenced in the activity of EO and Hy against each microbial strain. Table 6 shows the concentration of EOs and Hys necessary to obtain the Inhibitory concentration of the 50% (IC50) of the initial inoculum, and the IC50_Hy_/CF ratio that is the IC50_Hy_ value normalized according to the volatiles’ concentration. IC50 values were obtained, starting from the inhibition curve calculated using OD450 values obtained from the micro-broth dilution test. In Table 6, values of dermatophytes are not reported. In fact, due to the inhomogeneity of their growth, they were only evaluated by visual reading, as specified in “Material and Methods section”. Additionally, in this case, the visual exam points out that IC50_Hy_/CF ratios are significantly lower than the respective IC50_EO_ values.

More generally, the average values of IC50_Hy_/CF and IC50_EO,_ calculated on four bacterial strains (excluding 01SA(R) and 0.6EF strains) and four yeasts, indicate that the two distillation products (EO and Hy) from *S. montana* show the smallest differences in terms of effectiveness related to volatiles concentrations: in the average of the eight cases, an amount of the EO 8.2 times as concentrated as that of the Hy is needed to attain the same inhibition of microbial growth. However, products obtained from the *O. hirtum* and *Monarda* genus illustrate the greatest difference in terms of the biological activity related to the volatiles’ concentration. In fact, a quantity of *O. hirtum*, *M. didyma* and *M. fistulosa* EOs, respectively, 5.7, 16 and 42.3 times as concentrated as the corresponding Hys is necessary. In this respect, the IC50 comparison between EOs and Hys outlines the same ranking as the IR50 comparison between EOs and Hys (Table 5), strengthening the differences in efficacy between the two distillation products.

## 3. Discussion

For more than half a century, humans have relied primarily on antibiotics and vaccines to treat and prevent microbial infections. In recent decades, despite the great progress in the medical and pharmaceutical fields, the traditional treatment of infectious diseases is often ineffective due to the increased resistance of microbial strains to antibiotics. To date, one fifth of global deaths is due to infectious diseases [17], as the uncontrolled use of antibiotics in the clinical, veterinary, and agricultural fields has led to the spread of multidrug-resistant microbial strains. While the pharmaceutical industry has addressed this problem by modifying existing antibiotics and developing new ones, microbial strains respond to the pharmaceutical industry by inactivating these new strategies with the development of antibiotic resistance. This scenario clearly highlights the need for new antimicrobial agents with different modes of action than those of traditional antibiotics.

Natural products are among the most promising candidates because they have low toxicity, low environmental impact, and a broad spectrum of action when compared to synthetic antimicrobial substances.

Many studies have shown the antimicrobial activity of various EOs [18,19] also regarding muti-drug resistant bacteria and fungi, due to a broad spectrum of cytocidal activity [20,21]. For example, the EO of *S. montana,* in addition to anti-oxidant activity, proved effective against bacteria and dermatophytes; especially *T. violaceum*, *T. rubrum*, *T. tonsurans*, *T. mentagrophytes* and *P. oryzae* [22,23], while the EO obtained from *O. hirtum* showed antimicrobial activity against both Gram+ and Gram- strains [24,25]. The EOs belonging to the *Lavandula* genus, in addition to having an antimicrobial activity against a broad spectrum of microorganisms [26,27,28], show sedative properties on the central nervous system, as well as anti-inflammatory and re-epithelializing properties [29,30,31]. Furthermore, EOs and Hys derived from non-native plants belonging to the *Monarda* genus grown in Italy, have shown interesting antimicrobial activities towards Gram+, Gram- yeasts and environmental fungi [32,33,34].

The effectiveness of active ingredients was also studied. β-Linalool is a non-toxic alcohol most common in nature. It is present in the phytocomplexes of lavender EOs but also of many other EOs. In the EO of *Cinnamomum camphora* (Ho wood) it can reach concentrations higher than 90%. Literature data show its comprehensive range of bioactive properties including antimicrobial activity [35]. The main component of both EO and Hy of *O. hirtum* is the thymol, a phenol monoterpene isomer of carvacrol, particularly present in EOs obtained from species belonging to the *Thymus* genus. This natural compound has an antimicrobial spectrum wider than that of β-linalool, including Gram-positive, Gram-negative bacteria (especially pathogens of the airways), and fungi. Finally, it shows the ability to interfere with the fungal transformation process from the cellular form to the hyphal form [36]. The antimicrobial activity of carvacrol, main component of both *S. montana* and *Monarda* spp. natural products, is higher than that of the other volatile compounds due to the free hydroxyl group, hydrophobicity, and the phenol moiety. In particular, it shows a great activity against Gram- food-borne pathogens [37].

Among the main active compounds analyzed, it is possible to identify an activity gradient (linalool < thymol < carvacrol). This gradient is consistent with the data of antimicrobial efficacy actually observed, as the least active natural compounds are those obtained from the *Lavandula* genus, while the others show stronger antimicrobial activities.

Moreover, several EOs have been shown to interfere with the ability of microorganisms to form biofilm, which is often linked to chronic, difficult-to-treat infections such as skin and wound infections [38,39]. *S. montana* EO was shown to be able to inhibit biofilm formation and interfere with preformed biofilms of Gram+ bacteria, including *S. aureus* [23].

Despite the high antimicrobial activity of EOs, use as such is not recommended due to their high concentration of hydrophobic active ingredients with a toxic potential. Therefore, to avoid toxic effects, EOs need to be used in low concentrations by diluting them in an appropriate vehicle before use.

On the contrary, Hys are hydrophilic solutions containing up to a maximum 1g/L of the EOs active compounds. Although more perishable than EOs, they are generally safe and do not need to be diluted in a vehicle before use. This feature of Hys makes them interesting both for oral intake and skin applications. The latter use becomes especially important in the presence of skin infections.

However, the antimicrobial activity of Hys would certainly appear to be milder than that of the corresponding EOs. In fact, the simple comparison of MIC values obtained from the antimicrobial analysis of the EOs and Hys used in this study evidence that the first are more effective at a lower concentration. Table 1 and Table 2 show that the EOs active on at least the 50% of the strains have inhibitory and cytocidal actions at concentrations ranging between 0.125% *v*/*v* and 2% *v*/*v*. Whereas, the Hys must be used at concentrations between 25% *v*/*v* and 50% *v*/*v* to reach the same antimicrobial activity, i.e., they need to be from 25 to 200 times more concentrated than EOs.

However, if we consider the relative concentration of active chemicals, can we say that Hys really have milder antimicrobial actions than the corresponding EOs? Table 5 and Table 6 show that this cannot be said. In fact, the calculated IR50_Hy_/CF is lower than the IR50_EO_, as well as the IC50_Hy_/CF calculated for each microbial strain is lower than the IC50_EO_. This means that, to obtain the inhibition of 50% of growth of both the initial inoculum of each strain and total microbial strains, a concentration of EOs’ volatiles greater than that of the corresponding Hys is required. It results, therefore, in the Hys’ volatiles being relatively more effective than those of EOs. This activity could be due to the hydrophilic environment of Hy, which provides a greater bioavailability of volatiles for the interaction with bacteria and fungi [40], or to the antagonistic action present among chemical components of the EO phytocomplex.

These data are interesting because they show the antimicrobial activity of Hys from another point of view, especially as it concerns potential clinical applications for the treatment of skin infections. In fact, in these pathologies, local applications that are simultaneously effective for the patient and safe for intact or damaged skin are indispensable.

Potential applications encompass all small skin infections that need daily local treatments with antimicrobial creams and ointments, but also of more serious pathologies such as *Tinea capitis* generated by dermatophytes that essentially afflicts children, or antibiotic resistant/sensitive infections of sores or wounds whose treatment becomes important for skin re-epithelialization, or chronic vaginal infections induced by yeasts in which the topical use of concentrated EOs is absolutely contraindicated due to their toxicity.

In all cases, the use of Hys with antimicrobial activity compatible with a cutaneous or mucosal treatment would be of great interest. In fact, Hys are already on the market, and they can be used on the skin of non-allergic subjects without inducing adverse effects. Currently, Hys in Italy are used in formulations of cosmetic products for body care, or they are sold pure for cosmetic and food use. As is well known, the Italian market is a famous perfume and fragrance hub that is constantly looking for new products and is able to influence the Hys production of primary producers. Globally, the Hys market in Europe has been growing for several years, attaining, in 2018, a 40% share of the world market [41]. From 2019 to 2024, this share is set to increase by an additional 5.2% [42]. Owing to these reasons and in light of our preliminary data, it becomes more and more interesting to deepen the studies on Hys.

## 4. Materials and Methods

### 4.1. Clinical Strains

Fifteen clinical strains (six Gram-positive bacterial strains and nine fungal strains), which are potential skin pathogens provided by the UOC of Microbiology of Policlinico Universitario A. Gemelli of Rome, Italy, were used. Two of the six bacterial strains were resistant (R) to antibiotics. Bacterial strains were: *Staphylococcus aureus* MRSA (0.1R), *Streptococcus pyogenes* (0.2), *Enterococcus faecalis* VRE (0.3R), *Enterococcus faecium* (0.4), *Staphylococcus aureus* MSSA (0.5), *Enterococcus faecalis* (0.6). Whereas, four of the nine fungal strains were yeasts (*Candida albicans* (3.1), *Candida parapsilosis* (0.1R), *Candida glabrata* (0.2R), and *Candida tropicalis* (0.3R)), three of which were resistant to common antifungals, and five dermatophytes (*Trichophyton rubrum*, *Trichophyton tonsurans*, *Trichophyton soudanense*, *Trichophyton violaceum*, and *Microsporum canis*). Mueller Hinton medium (Becton Dickinson and Company, Cockeysville, MD, USA) was used to grow bacterial strains at 37 °C for 24 h, while fungal strains were grown on RPMI broth and Sabouraud agar medium (Oxoid, Wade Road, Basingstoke, Hants, UK). In particular, yeasts were grown at 37 °C for 24 h, and dermatophytes at 30 °C for 7 days.

### 4.2. Essential Oils and Related Hydrolates

EOs and Hy from six aromatic plants grown and processed in Italy were studied (*S. montana*, *L. angustifolia*, *L. intermedia*, *O. hirtum*, *M. didyma*, and *M. fistulosa*). All EOs and Hys were kindly granted by FX Laboratorio Benessere srl (Arzignano, Vicenza, Italy), except for those isolated from *M. didyma* and *M. fistulosa* species, which were provided by DISTAL, University of Bologna.

### 4.3. Gas Chromatography Mass Spectrometry Analysis

Analyses were performed on a Bruker ScionSQ gas chromatograph, coupled with a single quadrupole mass-spectrometer (GC-MS) (Bruker, Milan, Italy). Compounds were separated BD-5 a semi-standard non-polar column (30 m × 0.25 mm, i.d.0.25 μm) (Phenomenex, Bologna, Italy). EOs were diluted 1:1000 (*v*/*v*) in ethyl acetate, and 1 μL of this dilution was injected into GC-MS. Samples of hydrolate were diluted 1:5 (*v*/*v*) in ethanol (99.8%), and 1 μL of this dilution was injected into GC-MS. The percentage (*w*/*w*) of the amount of the compounds of EO present in Hy was carried out gravimetrically. Peaks were identified by comparing the retention times with those of authentic standard MS fragmentation patterns and final confirmation by matching with the components of the commercial library NIST mass spectral database (vers. 6.41). The percentage composition of the oils was computed by the normalization method from the GC peak areas. R.I. were generated by using a series of n-alkanes from C7 to C40 (Sigma-Aldrich, Milan, Italy) and compared with data reported in the literature [43,44,45,46]. All analyses were repeated in triplicate.

### 4.4. Gravimetric Analysis

Five mL of each Hy were subjected to liquid/liquid isolation with 5 mL of CH_2_Cl_2_ (*n* = 3). The organic phases were pooled, and the solvent evaporated by means of a rotary evaporator at reduced pressure. The residue obtained was weighed and the percentage (*w*/*v*) content of volatiles in the hydrolate evaluated.

### 4.5. Broth Microdilution Susceptibility Test

The broth microdilution (BMD) susceptibility test according to the European Committee on Antimicrobial Susceptibility Testing (EUCAST) international guidelines were performed. The BMD test was performed on a 96-well plate by adding 100 μL of a cell suspension equal to 5 × 10^5^ CFU/mL to a final volume of 200 μL. Scalar dilutions, between 50% *v*/*v* (500 μL/mL) and 3.125% *v*/*v* (31.25 μL/mL) of Hy and between 2% (20 μL /mL) and 0.06% (0.6 μL/mL) of EO were tested. EOs and Hys were dissolved in a suitable nutrient agar (as specified in paragraph 4.1) and 0.5% *v*/*v* of Tween 80 was used to deliver the EOs into the hydrophilic medium. Plates were incubated overnight at 37 °C. After this period, MIC values were determined by spectrophotometric reading at 450 nm (EL808, Biotek, Winooski, VT, USA), except for MICs values of the dermatophytes, which were assessed by visual reading. To evaluate the MLC, 5 μL of the content of each well was seeded on Muller Hilton or Sabouraud agar plates, which were incubated for 24 h at 37 °C. The MIC is defined as the lowest concentration that completely inhibits the organism’s growth when compared to the growth of control. Whereas, the MLC is defined as the lowest concentration corresponding to the death of 99.9% or more of the initial inoculum. Each test was performed in triple, and both negative and positive controls were included. Values corresponding to the IR or LR of 50% and 90% of all strains were calculated. As discussed in the “Data management” paragraph, the value corresponding to a concentration of EOs or Hys necessary to obtain the inhibition of 50% of the initial inoculum was extrapolated for each strain analyzed.

### 4.6. Comparison Between EO and Hy

Hy and EO comparison was made, as described in Di Vito M et al. [16]. Comparison was based on comparing the total volatiles content of EO with that of the corresponding Hy. Briefly, the Essential Oil Total volatiles Area (EOTA) and the Hydrolate Total volatiles Area (HYTA) were calculated by evaluating areas covered by the total volatiles in the chromatograms multiplied by EO and Hy respective dilutions prior to GC–MS (1000 and 5, respectively). The semi-quantitative volatiles’ Conversion Factor (CF) between the EO and the Hy was assumed to be the EOTA/HYTA ratio. Comparison between an EO and its corresponding Hy was made by dividing the IC50 or IR50 of each Hy by its CF. If the value of this ratio corresponds to the value of IC50 or IR50 of the EO, it means that the two natural products are equivalent in terms of relative antimicrobial activity, as the same amount of volatiles is needed in both EO and Hy to inhibit the growth of 50% of the initial inoculum. Whereas, values of this ratio lower or higher than the IC50 or IR50 of the OE show a relative antimicrobial activity of volatiles contained in the Hy higher or lower than that of the EO, respectively.

### 4.7. Data Management

The IC50 value of each natural substance (*O. hirtum, S. montana, M. didyma* and *M. fistulosa*) and distillation product (EO and Hy) vs. each microbial strain was obtained by interpolating the OD450 values corresponding to the tested dilutions with a regression line, and calculating the dilution value (% *v*/*v*) corresponding to half of the OD450 value of the positive control. All the values obtained from both the microbiological and chemical analyzes were processed obtaining mean and standard deviation values.

## 5. Conclusions

An intrinsic and intriguing question that emerges from this study is to establish which topical application (hydrophobic EOs or hydrophilic Hys) is most suitable for healing different skin infections. Our short communication highlights an aspect still unexplored by the scientific literature regarding the real antimicrobial effectiveness of the active ingredients contained in Hys compared to the EOs from the same plant source. The use of odorous aqueous solutions with low concentrations of active ingredients in the treatment of minor and chronic skin infections is certainly interesting for the fight against antibiotic resistance. Furthermore, since the terpenic active ingredients are not very soluble in water, most Hys have a low number still present; *O. hirtum*, has only one. This makes these natural products also interesting for pharmaceutical companies who are looking for new natural products with antimicrobial action, but need “standardizable” products to be tested in clinical trials conducted according to scientific rigor.

## Figures and Tables

**Table 1:** Chemical composition of EOs.

			Average (% *n* = 3)					
Components	E-RI	L-RI	*L. angustifolia*	*L. intermedia*	*O. hirtum*	*S. montana*	*M. didyma*	*M. fistulosa*
2,3-Dimethyl-3-buten-2-ol	741	746	-	-	-	0.05	-	-
Thujene	923	928	0.11	0.08	1.30	1.11	1.81	3.48
α-Pinene	931	936	0.29	0.63	0.76	0.76	0.57	0.79
Camphene	945	950	0.10	0.33	0.07	0.22	0.23	0.15
Sabinene	967	973	0.06	0.13	-	0.07	1.12	0.28
1-Octen-3-ol	974	980	0.08	0.08	0.10	0.47	4.50	4.08
3-Octanone	979	985	0.27	-	-	-	0.20	0.13
β-Pinene	972	978	0.14	0.51	0.09	0.11	0.27	0.26
Myrcene	983	989	3.56	1.39	1.12	0.95	2.28	3.62
α-Phellandrene	998	1004	0.10	0.04	0.23	0.21	0.40	0.66
Hexyl acetate	1004	1010	-	0.03	-	-	-	-
3-Carene	1005	1011	0.15	0.09	0.06	0.06	0.20	0.32
α-Terpinene	1011	1017	0.07	0.05	3.04	1.98	3.69	5.69
*p*-Cymene	1018	1024	0.13	0.05	18.83	9.82	8.08	13.85
Limonene	1024	1030	1.53	-	0.39	0.66	0.88	1.06
1,8-Cineole	1026	1032	1.54	9.20	0.04	0.20	1.36	-
(Z)-β-Ocimene	1031	1038	5.44	0.60	1.29	0.04	-	-
(E)-β-Ocimene	1041	1048	3.13	0.63	0.22	0.02	-	-
γ-Terpinene	1053	1060	0.19	0.12	23.81	13.44	13.07	16.85
*cis*- Linalool oxide (f)	1069	1075	0.13	0.06	-	-	-	-
Terpinolene	1080	1087	0.26	0.29	0.12	0.05	0.22	0.21
β−Linalool	1092	1099	28.36	27.99	0.40	0.48	8.71	1.24
No Match	1197	1203	0.04	-	-	-	-	-
1-Octen-3-ol, acetate	1103	1110	0.61	0.09	-	-	-	-
Neo-allo-ocimene	1122	1130	3.28	-	-	-	-	-
Camphor	1136	1143	0.25	7.27	-	-	-	-
*n*-Hexyl isobutyrate	1144	1151	-	0.05	-	-	-	-
Borneol	1159	1167	0.77	3.40	0.05	0.50	0.56	0.24
Lavandulol	1161	1168	0.14	-	-	-	-	-
*p*-Cymen-8-ol	1176	1184	-	-	-	0.01	-	-
Cryptone	1181	1189	0.11	-	-	-	-	-
α-Terpineol	1182	1190	0.31	0.50	0.06	0.06	0.94	0.23
*n*-Hexyl n-butyrate	1184	1192	0.23	-	-	-	-	-
*cis*-Sabinene hydrate	1212	1219	0.10	0.10	-	0.07	-	-
Isobornyl formate	1231	1239	0.03	-	-	-	-	-
Thymol methyl ether	1226	1234	-	-	5.37	-	4.47	0.40
Pulegone	1226	1234	-	-	4.05	-	-	-
Hexyl 3-methylbutyrate	1236	1244	-	0.07	-	-	-	-
Carvacrol methyl ether	1235	1243	-	-	-	-	7.36	6.74
Tymoquinone	1244	1252	-	-	-	0.03	-	-
Geraniol	1247	1255	-	-	-	-	-	0.47
Linalyl acetate	1247	1255	33.35	36.47	-	-	-	-
Bornyl acetate	1275	1284	0.07	-	-	-	-	-
Lavandulol acetate	1281	1289	1.28	2.31	-	-	-	-
Thymol	1282	1290	-	-	36.30	1.21	15.40	1.87
Carvacrol	1292	1300	-	-	0.13	63.16	20.59	35.18
*L*-Terpinen-4-ol	1295	1302	5.50	2.93	0.27	0.29	-	-
δ-Elemene	1328	1337	-	0.06	-	0.06	-	-
Neryl acetate	1354	1362	0.46	0.16	-	-	-	-
Carvacrol acetate	1364	1373	-	-	-	0.13	-	-
β-Copaene	1367	1376	0.04	-	-	0.04	-	-
α-Copaene	1367	1376	-	-	-	0.05	-	-
Geranyl acetate	1371	1380	0.78	0.30	-	-	-	-
β-Bourbonene	1375	1384	-	-	0.09	0.04	-	-
β-Elemene	1381	1390	-	-	-	0.01	-	-
Humulene	1397	1407	0.07	0.03	-	0.03	0.03	0.06
β-Caryophillene	1411	1420	5.75	1.71	0.67	1.53	1.00	1.20
*cis*-α-Bergamotene	1425	1430	0.22	0.09	-	-	-	-
*trans*-α-Bergamotene	1425	1434	0.05	0.05	-	-	-	-
*γ-Elemene*	1426	1436	-	-	-	0.09	-	-
(Z)-β-Farnesene	1436	1446	0.21	0.45	-	-	-	-
(E)-β-Farnesene	1446	1456	0.07	-	-	-	-	-
Geranyl propionate	1467	1477	-	0.24	-	-	-	-
γ-Muurolene	1466	1476	-	0.04	0.07	-	-	-
Germacrene D	1471	1481	0.21	0.28	-	0.28	-	-
Zingiberene	1485	1495	-	0.03	-	-	-	-
β-Bisabolene	1498	1508	-	-	0.41	0.88	-	-
γ-Cadinene	1503	1513	-	0.30	0.15	0.02	-	-
δ-Cadinene	1513	1523	0.04	-	0.23	0.07	-	-
β-Sesquiphellandrene	1513	1524	-	0.07	-	-	-	-
Caryophyllene oxide	1570	1581	0.08	-	-	0.07	-	-
Cadinol T	1629	1640	-	0.14	-	-	-	-
α-Bisabolol	1671	1683	-	0.14	-	-	-	-

Note. RI = Retention Indices. SD < 5%, RI-E = RI experimentally determined, RI-L = RI determined through Libraries.

**Table 2:** Chemical composition of volatile compounds in hydrolate.

			Average (%)					
Components	E-RI	L-RI	*L. angustifolia*	*L. intermedia*	*O. hirtum*	*S. montana*	*M. didyma*	*M. fistulosa*
3-Methyl-4-penten-1-ol	781	786	-	0.11	-	-	-	-
3-Hexen-1-ol	852	857	0.10	-	-	-	0.03	0.16
5,5-Dimethyl-2(5H)-furanone	946	952	0.52	-	-	-	-	-
1-Octen-3-ol	976	980	-	0.19	-	-	6.64	5.59
3-Octanone	979	985	-	-	-	-	0.05	0.04
1,8-Cineole	1026	1032	0.90	19.08	-	-	0.33	-
*cis*-Linalool oxide(f)	1069	1075	0.78	0.76	-	-	-	-
*trans*-Linalool oxide(f)	1077	1083	2.40	-	-	-	-	-
β-Linalool	1092	1099	42.15	34.17	-	-	6.94	0.63
Camphor	1136	1143	0.32	22.12	-	-	-	-
Eucarvone	1142	1150	0.15	-	-	-	-	-
Sabina ketone	1148	1156	0.14	-	-	-	-	-
Isopulegol	1152	1159	1.42	-	-	-	-	-
Borneol	1159	1166	2.50	3.17	-	-	0.77	0.22
α-Terpineol	1182	1190	19.01	5.20	-	-	1.56	0.30
Verbenone	1198	1206	-	-	-	0.05	-	-
Not identified	1209	1215	0.42	0.15	-	-	-	-
Cumin aldehyde	1230	1238	0.07	-	-	-	-	-
6,7-Dihydro-7-hydroxylinalool	1229	1237	3.58	1.17	-	-	-	-
2-Hydroxycineol	1239	1247	-	0.26	-	-	-	-
Geraniol	1247	1255	0.77	0.07	-	-	-	0.61
Thymol	1282	1290	-	-	100	13.88	34.03	6.66
Cumin alcohol	1282	1290	0.18	-	-	-	-	-
Not identified	1287	n.d.	0.52	-	-	-	-	-
Carvacrol	1292	1300	-	-	-	85.79	48.44	84.68
L-Terpinen-4-ol	1295	1302	20.23	7.63	-	-	1.22	1.11
Not identified	1406	n.d.	-	1.16	-	-	-	-
Not identified	1493	n.d.	2.99	-	-	-	-	-
Cadinol T	1629	1640	-	0.63	-	-	-	-
α-Cadinol	1641	1652	-	0.16	-	-	-	-
α-Bisabolol	1671	1682	-	0.77	-	-	-	-
Palmitic acid, ethyl ester	1981	1993	0.10	0.79	-	0.06	-	-
Stearic acid, ethyl ester	2183	2196	0.05	0.65	-	-	-	-
Squalene	2776	2790	0.03	1.44	-	0.21	-	-
Gravimetric analysis ^a^			0.09	0.05	0.04	0.06	0.03	0.04

Note. RI = Retention indices. ^a^ Values are expressed as % (*w*/*w*). SD < 5%, RI-E = RI experimentally determined, RI-L = RI determined through Libraries.

**Table 3:** Inhibitory and lethal activities of EOs.

		EOs (% *v*/*v*)											
	Clinical Strains	LA		LI		OH		SM		MD		MF	
D	Bacteria	MIC	MLC	MIC	MLC	MIC	MLC	MIC	MLC	MIC	MLC	MIC	MLC
0.1SA(R)	*S. aureus* MRSA	>2	>2	2	>2	≤0.06	<0.06	≤0.06	≤0.06	1	2	0.5	1
0.2SP	*S. pyogenes*	>2	>2	1	2	0.125	0.125	0.125	0.125	0.25	0.5	2	2
0.3EF(R)	*E. faecalis* VRE	>2	>2	2	2	0.125	0.125	0.125	0.125	0.25	0.5	2	2
0.4EF	*E. faecium*	>2	>2	2	2	0.125	0.125	0.125	0.125	0.5	0.5	2	1
0.5SA	*S. aureus* MSSA	>2	>2	2	>2	0.125	0.25	0.125	0.125	0.5	1	2	2
0.6EF	*E. faecalis*	>2	>2	2	2	≤0.06	0.25	≤0.06	0.125	0.25	>2	2	2
	Yeasts	MIC	MLC	MIC	MLC	MIC	MLC	MIC	MLC	MIC	MLC	MIC	MLC
3.1CA	*C. albicans*	>2	>2	2	>2	0.25	0.25	0.25	0.25	0.25	0.25	1	2
0.1CP (R)	*C. parapsilosis*	>2	>2	2	>2	0.25	0.5	0.25	0.25	0.5	0.5	1	2
0.2CG (R)	*C. glabrata*	>2	>2	2	>2	0.25	0.25	0.25	0.25	0.25	0.25	1	2
0.3CT (R)	*C. tropicalis*	>2	>2	2	>2	0.25	0.5	0.25	0.25	0.25	0.5	1	2
	Dermatophytes	MIC	MLC	MIC	MLC	MIC	MLC	MIC	MLC	MIC	MLC	MIC	MLC
*0.1TS*	*T. soudanense*	2	2	1	2	1	1	0.125	0.125	0.5	0.5	0.5	0.5
*0.2TS*	*T. tonsurans*	1	>2	1	2	0.5	0.5	0.125	0.125	0.25	0.5	0.25	0.25
*0.3TS*	*T. rubrum*	2	2	2	2	1	1	0.25	0.25	2	2	0.5	0.5
*0.4TS*	*T. violaceum*	0.125	0.06	0.125	0.06	0.125	0.06	0.125	0.125	0.25	0.125	0.125	0.06
*0.5TS*	*M. canis*	>0.5	>2	0.25	0.25	1	1	0.25	0.25	2	2	0.5	0.5
	IR90/LR90	>2	>2	2	>2	1	1	0.25	0.25	1	2	2	2
	IR50/LR50	>2	>2	2	2	0.25	0.25	0.125	0.125	0.25	0.5	1	2

Note. D = Designation, IR90= Inhibition Rate of 90% of strains, LR90 = Lethal Rate of 90% of strains, IR50 = Inhibition Rate of 50% of strains, LR50 = Lethal Rate of 50% of strains, LA = *Lavandula angustifolia,* LI=*Lavandula intermedia,* OH = *Origanum hirtum,* SM = *Satureja montana,* MD = *Monarda didyma,* MF = *Monarda fistulosa.*

**Table 4:** Inhibitory and lethal activities of Hys.

		Hys (% *v*/*v*)											
	Clinical Strains	LA		LI		OH		SM		MD		MF	
D	Bacteria	MIC	MLC	MIC	MLC	MIC	MLC	MIC	MLC	MIC	MLC	MIC	MLC
0.1SA(R)	*S. aureus* MRSA	>50	>50	>50	>50	6.25	50	>50	>50	>50	>50	>50	>50
0.2SP	*S. pyogenes*	>50	>50	>50	>50	50	>50	>50	>50	50	>50	>50	>50
0.3EF(R)	*E. faecalis* VRE	>50	>50	>50	>50	50	>50	>50	>50	50	>50	>50	>50
0.4EF	*E. faecium*	>50	>50	>50	>50	50	50	>50	>50	>50	>50	>50	>50
0.5SA	*S. aureus* MSSA	>50	>50	>50	>50	50	>50	>50	>50	50	>50	>50	>50
0.6EF	*E. faecalis*	>50	>50	>50	>50	50	>50	>50	>50	50	>50	>50	>50
	Yeasts	MIC	MLC	MIC	MLC	MIC	MLC	MIC	MLC	MIC	MLC	MIC	MLC
3.1CA	*C. albicans*	>50	>50	>50	>50	50	50	50	50	50	50	25	50
0.1CP (R)	*C. parapsilosis*	>50	>50	>50	>50	50	>50	50	>50	50	50	25	50
0.2CG (R)	*C. glabrata*	>50	>50	>50	>50	50	50	50	50	50	>50	25	50
0.2CT (R)	*C. tropicalis*	>50	>50	>50	>50	50	50	50	50	50	50	25	50
	Dermatophytes	MIC	MLC	MIC	MLC	MIC	MLC	MIC	MLC	MIC	MLC	MIC	MLC
*0.1TS*	*T. soudanense*	50	>50	50	>50	50	50	50	50	25	50	25	50
*0.2TS*	*T. tonsurans*	50	>50	50	>50	25	50	50	50	25	50	25	>50
*0.3TS*	*T. rubrum*	>50	>50	>50	>50	50	50	50	50	50	50	25	25
*0.4TS*	*T. violaceum*	50	50	12.5	25	6.25	12.5	25	25	12.5	12.5	≤6.25	6.25
*0.5TS*	*M. canis*	>50	>50	>50	>50	50	50	50	50	50	50	25	25
	IR90/ LR90	>50	>50	>50	>50	50	>50	>50	>50	>50	>50	>50	>50
	IR50/LR50	>50	>50	>50	>50	50	50	50	50	50	50	25	50

Note: D = Designation, IR90 = Inhibition Rate of 90% of strains, LR90 = Lethal Rate of 90% of strains, IR50 = Inhibition Rate of 50% of strains, LR50 = Lethal Rate of 50% of strains, LA = *Lavandula angustifolia,* LI = *Lavandula intermedia,* OH = *Origanum hirtum,* SM = *Satureja montana,* MD = *Monarda didyma,* MF = *Monarda fistulosa.*

**Table 5:** Volatile concentrations in EOs and HYs, their relationships, and IR50 comparison at equivalent volatile concentrations.

Heading	Total Area			
	*O. hirtum*	*S. montana*	*M. didyma*	*M. fistulosa*
EOTA	2.23 × 10^13^	7.13 × 10^13^	8.21 × 10^11^	7.53 × 10^11^
HYTA	2.03 × 10^10^	1.61 × 10^11^	5.03 × 10^8^	4.25 × 10^8^
CF	1.13 × 10^3^	4.42 × 10^2^	1.63 × 10^3^	1.77 × 10^3^
IR50_Hy_/CF (% *v*/*v*)	0.044	0.113	0.031	0.014
IR50_EO_ (% *v*/*v*)	0.25	0.125	0.25	1
IR50_EO_/(IR50_Hy_/CF)	5.68	1.11	8.33	71.43

Note: EOTA = Essential Oil Total volatiles Area, HYTA = Hydrolate Total volatiles Area, CF= volatiles’ Conversion Factor.

**Table 6:** Comparison of the IC50 values of each Hy vs. the corresponding EO.

		% v/v											
	Clinical Strains	OH			SM			MD			MF		
D	Bacteria	IC50_Hy_	IC50_Hy_/CF	IC50_EO_	IC50_Hy_	IC50_Hy_/CF	IC50_EO_	IC50_Hy_	IC50_Hy_/CF	IC50_EO_	IC50_Hy_	IC50_Hy_/CF	IC50_EO_
0.1SA(R)	*S. aureus* MRSA	1.94 ± 3.92	0.00 ± 0.00	n.c.	24.41 ± 2.60	0.06 ± 0.00	n.c.	52.77 ± 6.36	0.03 ± 0.00	1.17 ± 0.71	119.03 ± 17.50	0.06 ± 0.01	0.33 ± 0.04
0.2SP	*S. pyogenes*	31.23 ± 20.32	0.03 ± 0.02	0.14 ± 0.02	33.10 ± 1.80	0.07 ± 0.00	0.30 ± 0.17	28.87 ± 2.78	0.02 ± 0.00	0.36 ± 0.05	102.82 ± 54.31	0.06 ± 0.03	0.84 ± 0.01
0.3EF(R)	*E. faecalis* VRE	25.05 ± 9.28	0.02 ± 0.00	0.01 ± 0.02	38.27 ± 20.00	0.09 ± 0.04	0.10 ± 0.01	22.82 ± 0.22	0.01±0.00	0.18 ± 0.02	59.76 ± 10.71	0.03 ± 0.01	0.88 ± 0.01
0.4EF	*E. faecium*	21.67 ± 0.77	0.02 ± 0.00	0.04 ± 0.03	28.66 ± 3.12	0.07 ± 0.00	0.07 ± 0.01	35.28 ± 10.40	0.02±0.01	0.41 ± 0.04	40.88 ± 20.10	0.02 ± 0.01	0.41 ± 0.03
0.5SA	*S. aureus* MSSA	24.95 ± 10.50	0.02 ± 0.00	0.12 ± 0.02	29.78 ± 6.84	0.07 ± 0.01	0.10 ± 0.02	18.79 ± 0.26	0.01±0.00	0.45 ± 0.00	32.16 ± 14.41	0.02 ± 0.01	0.58 ± 0.03
0.6EF	*E. faecalis*	29.74 ± 3.98	0.03 ± 0.00	n.c.	68.10 ± 22.00	0.15 ± 0.05	n.c.	17.35 ± 0.01	0.01±0.00	0.21 ± 0.01	22.38 ± 7.69	0.01 ± 0.00	0.79 ± 0.03
	Yeast	IC50_Hy_	IC50_Hy_/CF	IC50_EO_	IC50_Hy_	IC50_Hy_/CF	IC50_EO_	IC50_Hy_	IC50_Hy_/CF	IC50_EO_	IC50_Hy_	IC50_Hy_/CF	IC50_EO_
3.1CA	*C. albicans*	11.60 ± 0.32	0.01 ± 0.00	0.15 ± 0.04	25.29 ± 4.57	0.06 ± 0.01	0.19 ± 0.01	27.57 ± 17.16	0.02 ± 0.01	0.01 ± 0.05	11.29 ± 5.04	0.01 ± 0.00	0.49 ± 0.04
0.1CP (R)	*C. parapsilosis*	20.75 ± 3.63	0.02 ± 0.00	0.16 ± 0.04	26.08 ± 1.86	0.06 ± 0.00	0.15 ± 0.01	20.78 ± 1.10	0.01 ± 0.00	0.13 ± 0.07	13.70 ± 0.24	0.01 ± 0.00	0.53 ± 0.04
0.2CG (R)	*C. glabrata*	27.53 ± 1.36	0.02 ± 0.00	0.22 ± 0.00	27.59 ± 0.92	0.06 ± 0.00	0.19 ± 0.01	28.92 ± 1.31	0.02 ± 0.00	0.29 ± 0.00	16.41 ± 0.03	0.01 ± 0.00	0.62 ± 0.11
0.3CT (R)	*C. tropicalis*	28.79 ± 2.24	0.03 ± 0.00	0.11 ± 0.03	24.33 ± 0.72	0.05 ± 0.01	0.18 ± 0.12	27.86 ± 3.87	0.02 ± 0.00	0.16 ± 0.01	15.14 ± 1.10	0.01 ± 0.00	0.38 ± 0.06

Note. n.c. = This value cannot be calculated because the corresponding MIC value is lower than the minimum dilution tested. IC50 = Inhibitory Concentration of the 50% of initial inoculum, CF = volatiles’ Conversion Factor, OH = *Origanum hirtum,* SM = *Satureja montana,* MD = *Monarda didyma,* MF = *Monarda fistulosa.*

## Data Availability

*M. didyma* and *M. fistulosa* plants were used in this study. Vouchers are deposited at the Herb garden of Casola valsenio (Ravenna, Italy) (Dr Sauro Biffi).
